# “When you are working in this environment, you’re more likely to get sick”: Mapping Care Relationships in LTC

**DOI:** 10.1093/geroni/igaa057.3413

**Published:** 2020-12-16

**Authors:** Andreina Marquez de la Plata Gregor, Katie Aubrecht, Tamara Daly, Ivy Bourgeault, Susan Braedley, Prince Owusu, Pat Armstrong, Hugh Armstrong

**Affiliations:** 1 St. Francis Xavier University, Antigonish, Nova Scotia, Canada; 2 York University, Toronto, Ontario, Canada; 3 University of Ottawa, Ottawa, Ontario, Canada; 4 Carleton University, Ottawa, Ontario, Canada

## Abstract

The pandemic has shone a light on problems within the long-term care (LTC) sector. As was true prior to COVID-19, many of the present issues in LTC can be traced to challenging working conditions, such as persistent understaffing of care workers. Working short-staffed means rushing through care, while only satisfying the most basic bodily needs of the resident. This presentation shares early findings from a thematic analysis of interviews conducted with seven care workers as part of the “Mapping Care Relationships” stream of the Seniors –Adding Life to Years (SALTY) project, a pan-Canadian research program that maps how promising approaches to care relationships are organized and experienced in LTC. The purpose of the analysis was to understand how short-staffing is affecting the formation and preservation of meaningful staff-resident relations, and what the impact is on quality of care. Two overarching themes emerged: 1) a relationship between time and work-place illness, injury and violence; 2) a relationship between care worker autonomy and resident quality of care. When working conditions do not support workers in voicing and/or addressing challenges they experience in the workplace, whether this results from understaffing or hierarchical power structures, care workers’ ability to deliver even basic care is jeopardized, and resident and worker health and wellness are placed at risk. Themes are discussed in the context of COVID-19 in light of responses to outbreaks in LTC that have reduced the availability of care workers, family visitors and volunteers, and emphasized top-down and even militarized approaches to care management.

